# Optimization of the adipose-derived mesenchymal stem cell delivery time for radiation-induced lung fibrosis treatment in rats

**DOI:** 10.1038/s41598-019-41576-5

**Published:** 2019-04-03

**Authors:** Yang Zhang, Xinping Jiang, Liqun Ren

**Affiliations:** 1 Department of Experimental Pharmacology and Toxicology, Pharmaceutical Science of Jilin University, Changchun, Jilin 130021 China; 2 Department of Vascular Surgery, The first Hospital of Jilin University, Changchun, Jilin 130021 China; 3 Department of Oncological Radiotherapy, The First Hospital of Jilin University, Changchun, 130021 China

## Abstract

The present study attempts to identify the optimal time duration for the administration of Ad-MSCs, in order to maximize its therapeutic benefits, and compare the degree of fibrosis among three different administration time points using the RILF rat model system. Ad-MSCs were delivered to Sprague-Dawley rats through the tail vein at the following different time points after thorax irradiation: two hours, seven days, and two hours + seven days. Post Ad-MSCs transplantation and the histopathological analysis of the lungs were performed along with analysis of inflammatory cytokine levels, including interleukin (IL)-1, IL-2, IL-6, IL-10 and tumor necrosis factor-α (TNF-α). In particular, pro-fibrotic factors (TGF-β1 and α-SMA) were also evaluated in serum and lung tissues. In addition, it was also determined whether Ad-MSCs had any role in inhibiting the transition of type II alveolar epithelial cells into fibroblasts in the lungs of injured rats. The present results demonstrated that the intravenous delivery of Ad-MSCs twice at the 2-hour and 7-day (R + MSC_2h+7d_ group) was effective in reducing lung fibrosis for long term durations, when compared with single delivery either at the two-hour or 7-day time points. In addition, a marked anti-inflammatory effect was also observed in RILF rats in the R + MSC_2h+7d_ group, as indicated by the reduced serum levels of pro-inflammatory cytokines (TNF-α, IL-1 and IL-6) and increased levels of anti-inflammatory cytokines IL-10 and IL-2. Rats that were delivered twice with Ad-MSCs (R + MSC_2h+7d_ group) exhibited significantly reduced TGF-β1 and α-SMA levels, in contrast to rats in the R + MSC_7d_ or R + MSC_2h_ groups, after four weeks. Furthermore, it was also noted that after four weeks, Ad-MSCs increased the number of lung epithelial cells (SP-C) and inhibited the lung fibroblastic cells (α-SMA) of rats in the R + MSC_2h_ and R + MSC_2h+7d_ groups. The present study concluded that two injections of Ad-MSCs (R + MSC_2h+7d_ group) appear to be optimal for therapeutic efficacy and safety during RILF.

## Introduction

Radiation-induced lung fibrosis (RILF) represents a common and major complication due to radiotherapy, and thereby presents a threat to the health and life of patients^1,2^. The RILF clinical incidence rate ranges within 10–20%^3^. It appears to be a complex pathological process with excessive secretion of various cytokines, which subsequently enhances fibroblast replication and extracellular matrix (ECM) deposition, thereby resulting in impaired lung function^4,5^.

Among various cytokines, transforming growth factor-β1 (TGF-β1), an important pro-fibrotic growth factor, has been linked with induction of lung fibrosis^6,7^. Furthermore, it has been presumed to be required for the generation of fibrotic foci^8–10^. An increasing body of evidence has also highlighted the importance of TGF-β1 in inducing epithelial-to-mesenchymal transition (EMT), which is typically characterized by loss of cell adhesion, the *de novo* expression of α-SMA, and other fibrogenic mediators^9–11^. The TGF-β1-dependent induction of myofibroblasts from lung epithelial cells has been an important step towards fibroblastic foci formation in RILF^12^.

Mesenchymal stem cells (MSCs) have been shown to be clinically important^13–15^. Multiple studies have indicated the beneficial effects of adipose-derived mesenchymal stem cells (Ad-MSCs) in injured lung tissues^16,17^. The possible mechanisms of MSC-based lung repair are not just limited to releasing autocrine or signals. Other mechanisms, such as anti-oxidation and mitochondria transfer of MSCs also contribute to the lung repair^18,19^. Since adult adipose tissue appears to be an abundant, accessible and safe source of stem cells, Ad-MSCs can prove to be promising for stem cell therapy against lung disease^20–22^. However, the optimal strategy for its clinical application has not yet been identified or characterized, and the dosing schedules along with therapeutic effects and safety remain controversial at present. Importantly, other studies have indicated that the irradiation-mediated stimulation of TGF-β1 release peaked at two and four weeks within a 24-week experiment period^23^. Thus, based on the clues obtained from these studies, it was proposed that the double intravenous delivery of Ad-MSCs, depending on the TGF-β1 expression, might be helpful in RILF treatment. Therefore, in the present study, the investigators attempted to identify the optimal time duration for Ad-MSC administration, in order to maximize the therapeutic benefits. In addition, the degree of fibrosis during three different administration times was also compared using the RILF rat model system by analyzing inflammatory factors and TGF-β1 levels through its downstream effects on lung cell injury.

## Materials and Methods

### Procurement of animals and ethical approval for the study

Sprague-Dawley (SD) male rats, weighting approximately 220–250 g, were purchased from the Laboratory Animal Center of the Academy of Military Medical Sciences (Beijing, China). All experiments that involved animal subjects were performed according to the guidelines approved by the ethics committee of the Pharmaceutical Science of Jilin University.

### Radiation schedule and experimental design

A total of 250 male adult SD rats were randomly divided into five groups: (1) control group; (2) RILF group; (3) RILF + MSC_2h_ group; (4) RILF + MSC_7d_ group; (5) RILF + MSC_2h+7d_ group. Each group had 50 rats. The right thorax of each rat was irradiated using a single-dose radiation of 15 Gy with X-rays, while the rest of the body was shielded with lead strips. After thorax irradiation, the animals received 5 × 10^6^ of Ad-MSCs *via* the tail vein at each time point. Subsequently, the peripheral blood and lung tissue samples were collected from each rat at different times points corresponding to the latent period (at 1, 2, 6, 12, 24, 48 and 72 hours, and at one week post-irradiation), the pneumonic phase (at 2, 4, 8 and 12 weeks post-irradiation), and at the beginning of the fibrotic phase (at 20 weeks post-irradiation) for further analysis. The irradiated rats that received phosphate buffered saline (PBS) were assigned as controls.

### Isolation, culture and characterization of Ad-MSCs

Ad-MSCs were isolated from adipose tissues obtained from control and experimental rats, according to a previously described protocol^24^. The isolated MSCs after passage three were used for injection in all experiments. Cells were tested for cell surface markers using anti-rat *CD73-PE, CD90-PE and CD105-PE* antibodies. Mouse *lgG1-FITC* and *PE* were used for isotype control. All antibodies were purchased from eBioscience (San Diego, CA, USA). Adipogenic, chondrogenic and osteogenic differentiation assays were performed using the respective induction medium under standard culture conditions, according to manufacturer’s protocol (Trevigen). Thereafter, every 2–3 days, the medium was changed. After 21 days, the cells were harvested for further analysis. In order to identify adipocytes, the oil red O staining of intracellular fat droplets was performed. Similarly, in order to identify osteoblasts, calcium deposits in cells were stained by Alizarin Red, while chondrocytes were identified using collagen staining with toluidine blue dye.

### Histopathology

The isolated rat lung tissues were fixed with 4% paraformaldehyde, and embedded in paraffin sections of 5-μm average thickness. The sections were stained with hematoxylin and eosin (H&E) and Masson’s trichrome. The staining intensity of each section was semi-quantitatively analyzed by two blinded observers using five random images per group.

### Enzyme-linked immunosorbent assay (ELISA)

Serum was collected from the peripheral blood of each rat by centrifugation, and the concentration levels of TGF-β1 and α-SMA were measured using ELISA kits (R&D Systems), according to the manufacturer’s instructions.

### Immunohistochemistry

The paraffin-embedded lung sections were first dewaxed and rehydrated. Subsequently, the antigen was retrieved. Then, the sections were incubated in 0.3% H_2_O_2_ to block the endogenous peroxidase activity. Afterwards, the nonspecific antigen-binding sites were blocked by incubation with serum from the host. Next, the sections were incubated with specific primary antibodies (TGF-β1 [Abcam, Cambridge, MA, USA] and hydroxyproline [Abcam, Cambridge, MA, USA]) overnight at 4 °C. These specific primary antibodies were diluted according to manufacturer’s recommendations.

At the following day, relevant secondary antibodies were added, and the sections were further incubated at 37 °C for two hours. Then, the DAB solution was added to detect positive cells. In addition, an immunohistochemistry (IHC) staining kit (Abcam) was used to detect the expression of TGF-β1 and hydroxyproline.

### Immunofluorescence

After dewaxing, rehydration and antigen retrieval, the paraffin-embedded rat lung sections were permeabilized with 0.3% Triton X-100 for 30 minutes, and blocked with 3% BSA for another 30 minutes. The primary antibodies (proSP-C and α-SMA) used for immunofluorescence (IF) were obtained from Abcam (Cambridge, MA, USA). Alexa Fluor 488 and Alexa Fluor 594 (Invitrogen) were used as secondary antibodies. The nuclei were stained for three minutes with 4′,6-diamidino-2-phenylindole (DAPI), which was procured from Invitrogen. For each sample, five random fields were randomly captured. The fluorescence density of α-SMA was quantified using the LAS3000 apparatus (Fujifilm, Raytest, Courbevoie, France).

### Real-time PCR analysis

Total RNA from 100 mg of irradiated lung tissue obtained from each rat was freshly isolated using TRIzol reagent (Invitrogen). Then, 1 μg of total RNA obtained from each sample was used for the synthesis of first-strand cDNA using a RT-PCR Kit (Takara Bio Inc., Shiga, Japan). Next, the total cDNA was mixed with the forward and reverse primers of *TGF-β1, α-SMA, TNF-α, IL-1β, IL-2, IL-6 and IL-10* genes, and quantitative PCR amplification was performed using SYBR Green I TaqMan probes (Roche, Basel, Switzerland) in 40 amplification cycles in an ABI 7500 Fast machine. The expression levels were finally normalized to β-actin expression. All reactions were carried out in duplicate, and the results were analyzed using the 2^−∆∆CT^ method. The primer sequences used in the present study were as follows:

TGF-β1: Forward 5′-GAGAGCCCTGATACCAACGATCT-3′

Reverse 5′-GTGTGTCCAGGCTCCAAATGTAG-3′;

*α-*SMA: Forward 5′-GGCATCCACGAAACCACCTA-3′

Reverse 5′-TGAAGGCGCTGATCCACAAA-3′;

TNF-*α*: Forward 5′-ATCCGCGACGTGGAACTG-3′

Reverse 5′-ACCGCCTGGAGTTCTGGAA-3′;

IL-1β: Forward 5′-GAAATGCCACCTTTTGACAGTG-3′

Reverse 5′-TGGATGCTCTCATCAGGACAG-3′;

IL-2:  Forward 5′-TGAGCAGGATGGAGAATTACAGG-3′

Reverse 5′-GTCCAAGTTCATCTTCTAGGCAC-3′;

IL-6:  Forward 5′-CTGCAAGAGACTTCCATCCAG-3′

Reverse 5′-AGTGGTATAGACAGGTCTGTTGG-3′;

IL-10:  Forward 5′-CTTACTGACTGGCATGAGGATCA-3′

Reverse 5′-GCAGCTCTAGGAGCATGTGG-3′;

β-actin: Forward 5′-TTCTTTCTACAATGAGCTGGTGGC-3′

Reverse 5′-CTCATAGCTTCTGCAGGGAGGA-3′

### Western blot analysis

For the western blot analysis, lung tissues were homogenized in an appropriate amount of ice-cold lysis buffer. Equivalent amounts of protein (25–40 μg) were separated by using 15% sodium dodecyl sulfate polyacrylamide gel electrophoresis and transferred onto a polyvinylidene fuoride membrane (Millipore). Then, the membrane was probed with the *α-*SMA antibody (1:750, Abcam, Cambridge, MA, USA). β-actin (Kangchen) was used as an internal control. Immunoreactivity was detected using the Odyssey Infrared Imaging System (Gene Company Limited). All blots were exposed for optimal lengths of time for visualization. The band intensity was quantified using the LAS3000 apparatus (Fujifilm, Raytest, Courbevoie, France).

### Statistical analysis

All statistical analyses were performed using SPSS 19.0 software (IBM Corp., Armonk, NY, USA) and expressed as mean ± standard deviation (SD). Comparisons among groups were performed using one-way ANOVA. Statistical significance was defined as a *P*-value of ≤0.05.

## Results

### Characterization of isolated Ad-MSCs

**Figure 1:**
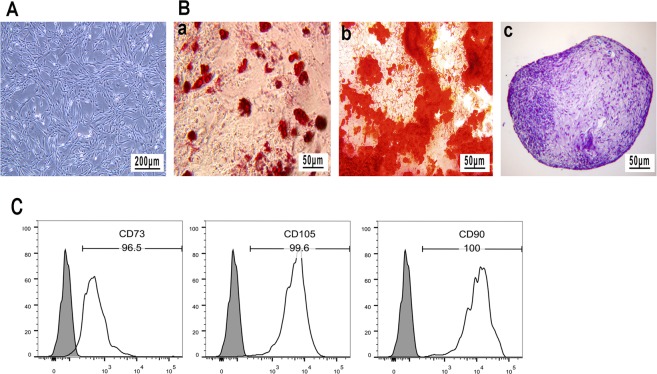
Characterization of rat Ad-MSCs. (**A**) Undifferentiated rat Ad-MSCs. Scale bar: 200 μm. (**B**) Rat Ad-MSCs were differentiated into (a) adipocytes (b) osteoblasts and (c) chondrocytes, as assessed by Oil Red O, Alizarin Red and toluidine blue staining, respectively. (**C**) The analysis of cell surface markers by fluorescence-activated cell sorting (FACS) assay.

The MSCs isolated from the adipose tissue of rats depicted a spindle-like morphology. The multipotency of rat Ad-MSCs was further confirmed by their differentiation into adipocytes, osteoblasts and chondrocytes after 21 days of culture in the defined culture media (Fig. 1A,B). In particular, the isolated Ad-MSCs typically revealed a positive staining for CD73 (96.5%), CD90 (100%) and CD105 (99.6%) markers (Fig. 1C). These results demonstrate that these cells satisfy the minimal criteria to be identified as MSCs *in vitro*^25^.

### Attenuated histopathology of Ad-MSCs in the RILF model

**Figure 2:**
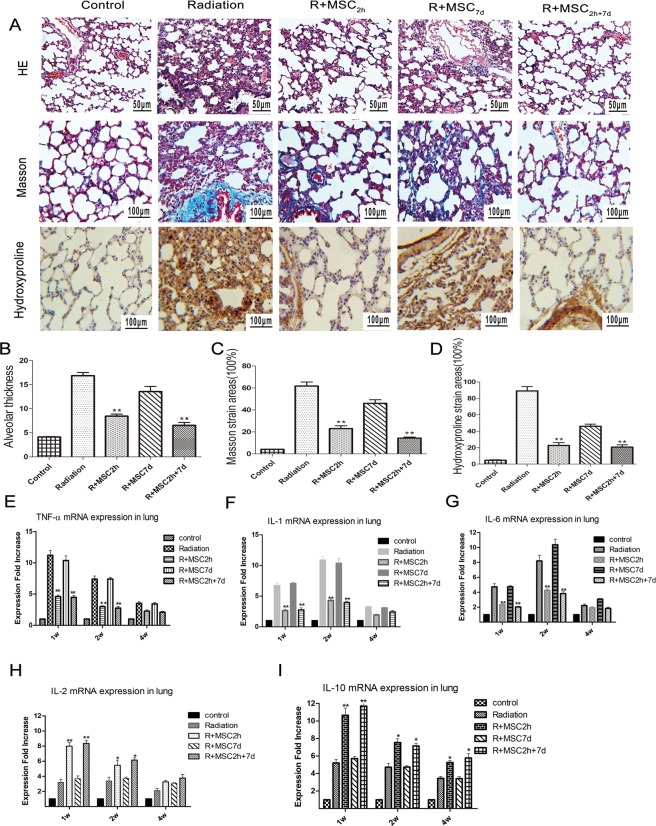
Histological changes and analysis of inflammatory cytokines in irradiated lung tissues within four weeks. (**A**) H&E, Masson’s and hydroxyproline staining analysis of lung sections at four weeks post-irradiation. Magnification: ×200; Scale bar: 100 μm; for H&E staining. Magnification: ×400; Scale bar: 50 μm; for Masson’s staining and hydroxyproline. (**B**–**D**) Semi-quantitative assessment of alveolar thickness, Masson’s stained areas and hydroxyproline staining areas after the analysis of five random images per group. (**E**–**I**) The mRNA expression of pro-inflammatory cytokines (TNF-α, IL-1β and IL-6) and anti-inflammatory cytokines (IL-2 and IL-10) were tested using quantitative real-time PCR. The expression levels were finally normalized to β-actin expression. All reactions were carried out in duplicate, and the results were analyzed using the 2^−∆∆CT^ method. Data are expressed as mean ± standard deviation (*n* = 3) and analyzed by independent samples *t*-test (*P ≤ 0.05 *vs*. the radiation group; **P ≤ 0.01 *vs*. the radiation group).

In addition, the histological changes in the lung sections of rats obtained from different treatment groups were also assessed after four weeks of thoracic irradiation by H&E, Masson’s and hydroxyproline staining (Fig. 2A). The lung section obtained from control rats exposed to radiation revealed radiation-induced inflammatory infiltrates, alveolar hemorrhage, disruption of the alveolar wall, and thickening of the interalveolar septum at the 4^th^ week. In contrast, the lung section of rats exposed to radiation along with Ad-MSC treatment revealed a significant reduction in airspace inflammation, alveolar hemorrhage and thickening of the interalveolar septa in both R + MSC_2h_ and R + MSC_2h+7d_ groups, while a slight reduction was observed in rats from the R + MSC_7d_ group. Moreover, Masson’s and hydroxyproline staining revealed a marked deposition of collagen in the lung tissue section from rats exposed to radiation after four weeks. However, the Ad-MSC treatment of rats in the R + MSC_2h_ and R + MSC_2h+7d_ groups resulted in reduced collagen deposition, when compared to rats from the R + MSC_7d_ group and radiation alone group. The semi-quantitative analyses of the histological observations are presented in Fig. 2B–D. It is noteworthy that the lung damage scores had no statistical difference between rats in the R + MSC_2h_ and R + MSC_2h+7d_ groups, and the scores of both groups were higher, when compared to the scores of the R + MSC_7d_ group and radiation-alone group (**P ≤* 0.01*).

### Ad-MSC treatment reduced pro-inflammatory and increased anti-inflammatory cytokine levels

The mRNA levels of pro-inflammatory cytokine TNF-α, IL-1β and IL-6 in lung tissues were also measured by real-time PCR analysis at 1, 2 and 4 weeks after irradiation. Irradiation markedly increased inflammatory cytokine TNF-α, IL-1β and IL-6 gene expression. Interestingly, Ad-MSC_2h_ or Ad-MSC_2h+7d_ treatment significantly reduced the irradiation-mediated release of TNF-α, IL-1 and IL-6, when compared to lungs obtained from rats in the radiation and R + MSC_7d_ groups during the first four weeks post-irradiation, as shown in Fig. 2E–G (P ≤ 0.01). The simultaneous analysis of anti-inflammatory cytokine IL-10 and IL-2 mRNA levels revealed that these significantly increased in both Ad-MSC_2h_ and Ad-MSC_2h+7d_ treatments, when compared with the radiation group or Ad-MSC_7d_ group, at the first week post-irradiation (*P* ≤ *0.01*).

### Lung radiation-increased profibrotic factor TGF-β1 production

**Figure 3:**
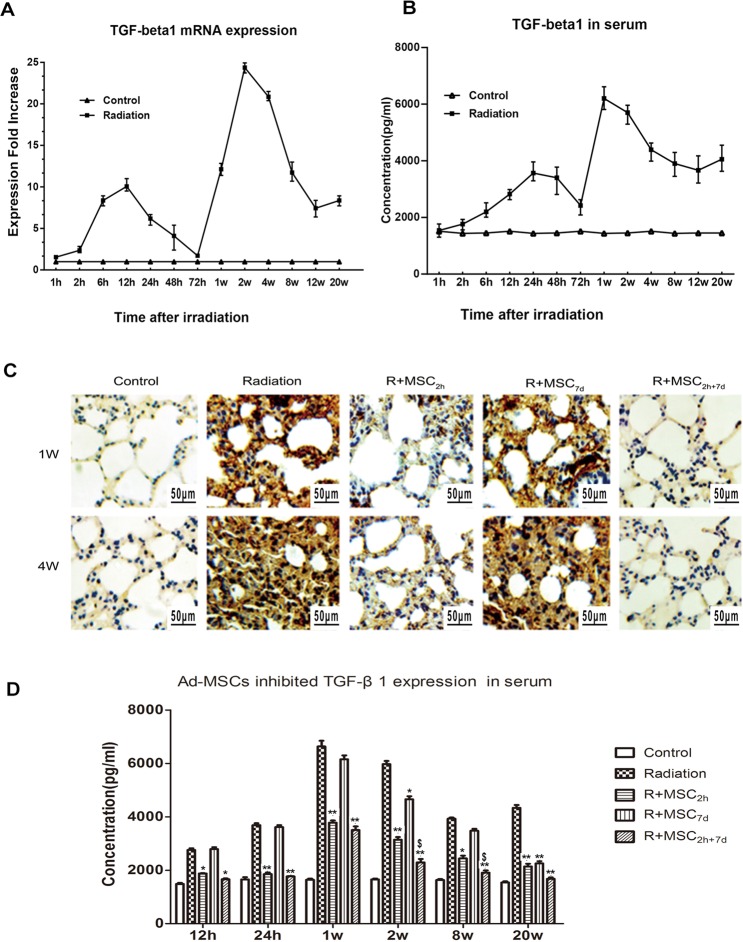
Lung radiation increased pro-fibrotic factor TGF-β1 production, and Ad-MSCs inhibited its levels in the lungs. (**A**) RT-PCR-based estimation of TGF-β1 mRNA expression in lung tissues at different time points (1, 2, 6, 12, 24, 48 and 72 hours, and 1, 2, 4, 8, 12 and 20 weeks) after 15 Gy of thoracic irradiation. (**B**) Serum TGF-β1 levels as detected using enzyme-linked immunosorbent assay (ELISA) kits, according to manufacturer’s instructions, during the 20-week time period. Control: TGF-β1 expression in serum and lung tissues in non-irradiated lung tissues of control animals. Three rats per time point were analyzed; each dot represents the mean of two measurements. (**C**) Assessment of TGF-β1 expression using the IHC method at one and four weeks. Magnification: ×400. Scale bar: 50 μm. (**D**) The analysis of TGF-β1 expression in serum samples using the ELISA method at different time points at post-irradiation. Data were presented as mean ± standard deviation (*P ≤ 0.05 *vs*. the radiation group,**P ≤ 0.01 *vs*. the radiation group; ^$^*P* ≤ 0.05*vs*. R + MSC_2h_ group, ^$$^*P* ≤ 0.01 *vs*. the R + MSC_2h_ group).

The TGF-β1 expression changes in the lung tissue and serum obtained from rats exposed to thoracic radiation alone (RT-alone group) were analyzed, as shown in Fig. 3. The TGF-β1 expression in lung tissues was observed to be elevated in a biphasic manner after thoracic irradiation. Specifically, a single dose of 15 Gy of radiation induced TGF-β1 mRNA very early, and the first peak (9.74-fold increase) was noticed after 12 hours. Subsequently, the TGF-β1 mRNA levels decreased, and the second peak (24.3-fold increase) was observed after 14 days of radiation exposure, as shown in Fig. 3A. In contrast, the mRNA isolated from controls with untreated rat lung tissues exhibited a very low TGF-β1 mRNA expression. In addition, the radiation-induced TGF-β1 levels in the serum of rats in the RT alone group reached its highest level within seven days, and subsequently gradually declined. However, the maximal level of TGF-β1 in serum was observed after 20 weeks of irradiation (Fig. 3B).

### Ad-MSCs inhibited the thoracic radiation-mediated increased in TGF-β1 production in lungs

In further investigating the effects of Ad-MSCs at different dosing times, on the radiation-induced TGF-β1 expression, a dramatic reduction in TGF-β1 expression was observed in the two Ad-MSC treatment groups. Particularly, the injection of Ad-MSCs within two hours and two hours + seven days of thoracic irradiation significantly inhibited the radiation-enhanced TGF-β1 expression in lung tissue samples after two and four weeks, as shown in Fig. 3C (P ≤ 0.01). A similar effect on TGF-β1 levels was also observed in serum after 12 hours, and this continued up to 20 weeks in the Ad-MSC_2h_ and Ad-MSC_2h+7d_ treatments, among all groups in Fig. 3D. However, serum TGF-β1 levels markedly decreased in the Ad-MSC_2h+7d_ group, when compared to the Ad-MSC_2h_ group, at two weeks (*P* ≤ *0.05*). However, the injection of Ad-MSCs at the 7^th^ day did not exhibit a similar effect on TGF-β1 expression, both in lung tissues and serum samples.

### Ad-MSC treatment inhibited α-SMA expression in lung tissue samples

**Figure 4:**
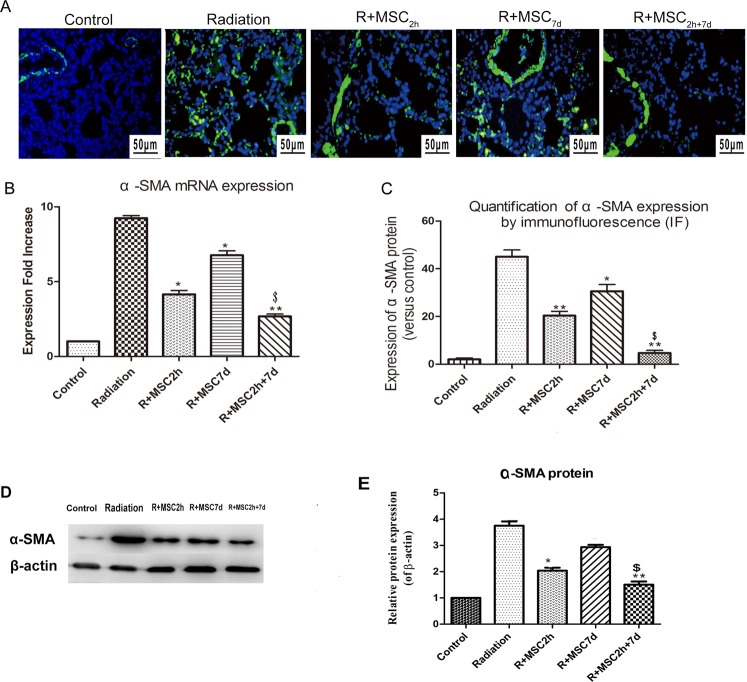
Analysis of α-SMA expression in lung tissues. (**A**) The assessment of α-SMA expression by immunofluorescence (IF) in lung tissue sections obtained from controls and the different treatment groups after four weeks of irradiation. Magnification: ×400. Scale bar: 50 μm. (**B**) The mRNA α-SMA expression analysis after four weeks of thoracic irradiation. β-actin expression was used as an internal control, and fold expression values were normalized to controls. (**C**) The quantification of α-SMA protein expression, as detected by IF after four weeks, and the analysis of five random images per group. The fluorescence intensity of α-SMA was quantified using an LAS3000 apparatus (Fujifilm, Raytest, Courbevoie, France). (**D**) The protein expression levels of α-SMA in lung tissues were analyzed by western blot after four weeks. The representative bands are shown. Full-length gels and blots are included in the Supplementary Fig. 1. (**E**) The density values of blots were normalized to the internal control, β-actin. The band intensity was quantified using the LAS3000 apparatus (Fujifilm, Raytest, Courbevoie, France). Data were presented as mean ± standard deviation (*P ≤ 0.05 *vs*. the radiation group, **P ≤ 0.01 *vs*. the radiation group; ^$^*P* ≤ 0.05 *vs*. the R + MSC_2h_ group, ^$$^*P* ≤ 0.01 *vs*. the R + MSC_2h_ group).

In parallel, the protein expression of α-SMA in lung tissue sections was also assessed after AD-MSC delivery by IF staining and western blot at four weeks, as shown in Fig. 4. As observed in Fig. 4A,D, the radiation-induced α-SMA protein expression in injured rat lung sections was significantly attenuated due to Ad-MSC treatment in the R + MSC_2h_ and R + MSC_2h+7d_ groups (*P* ≤ *0.01*). However, protein TGF-β1 levels markedly decreased in the Ad-MSC_2h+7d_ group, when compared to the Ad-MSC_2h_ group, at four weeks (*P* ≤ *0.05*). In parallel, the mRNA analysis at four weeks post-irradiation also revealed a significant increase (8.63-fold increase) in α-SMA expression in rat lung tissues. However, these levels were reduced dramatically in rats injected with Ad-MSCs in the R + MSC_2h+7d_ group at four weeks post-irradiation (*P* ≤ *0.01*). Furthermore, a 6.74-fold expression was still observed in rats in the R + MSC_7d_ group. In addition, rats in the R + MSC_2h+7d_ group had intermediate levels of decrease in α-SMA mRNA expression levels, when compared to the R + MSC_2h_ group (*P* ≤ *0.05*, Fig. 4B).

### Ad-MSCs reduced type II alveolar epithelial cell transition to fibroblastic phenotype

**Figure 5:**
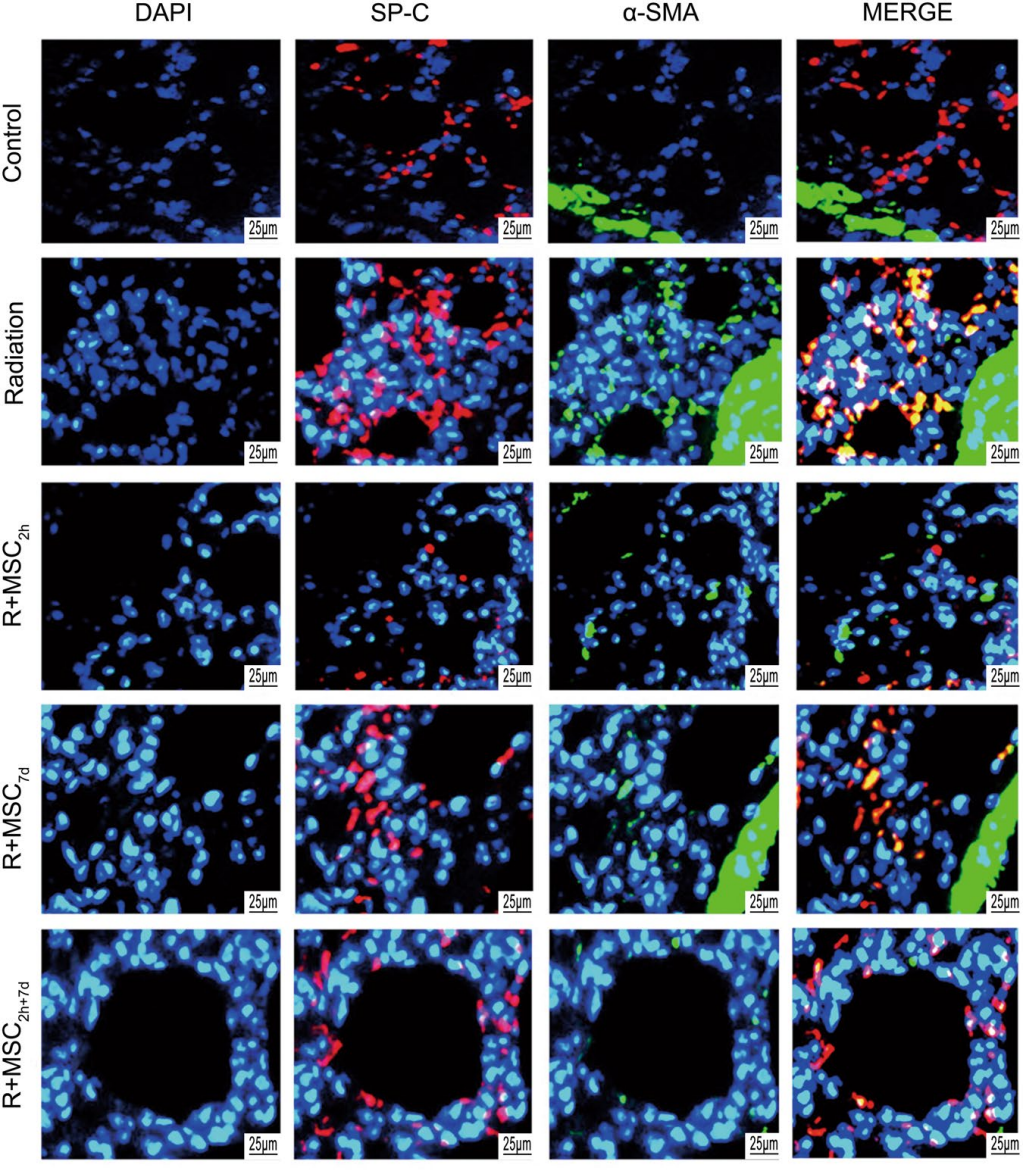
Effect of Ad-MSCs on type II alveolar epithelial cell transition into the fibroblastic phenotype. The SP-C and α-SMA staining analyses in lung sections obtained from rats in the control and treatment groups after four weeks of thoracic irradiation. DAPI staining was used to stain the nucleus. Magnification: ×800. Scale bar: 25 μm (DAPI, blue; proSP-C, red; α-SMA, green).

Finally, it was also determined whether Ad-MSC treatment has any role in inhibiting the transition of type II alveolar epithelial cells into the fibroblastic phenotype in injured rat lungs. This was conducted by analyzing the expression of type II alveolar epithelial cell marker, pro-surfactant protein C, and pro-fibrotic marker, α-SMA (Fig. 5). The present results indicated that exposure to radiation induced the transition of type II alveolar epithelial cells into the fibroblastic phenotype. However, Ad-MSC treatment induced a general reduction in the expression of pro-fibrotic marker α-SMA. In particular, the lung section obtained from rats in the R + MSC_2h+7d_ group exhibited a marked downregulation in α-SMA signal.

## Discussion

The present study confirmed the hypothesis that the injection of Ad-MSCs within two hours and one week of thoracic irradiation resulted in beneficial outcomes in the treatment of RILF, when compared to one late injection after seven days (R + MSC_7d_ group) or the radiation alone group.

Sprague-Dawley rats were given a single 15-Gy dose of irradiation to the right thorax to successfully establish the model of RILF in rats. Previous evidence has shown that the delivery of a minimum number of Ad-MSCs resulted in great therapeutic effects in irradiated lungs^26,27^. Xia *et al*. revealed that low-dose MSCs had greater safety and better therapeutic effects for radiation lung injury, when compared with high-dose MSCs^28^. On the contrary, a higher incidence of adverse events may occur in the high-dose group. Li *et al*. revealed that high-dose MSCs induced lethal portal vein embolization in mice with liver disease^29^. Previous animal experiments and clinical trials studies have shown that the injection of numbers between 1 × 10^3^/g and 5 × 10^4^/g was considered as safe dose for the treatment of lung injury^30,31^. The previous study conducted by the investigators also demonstrated that the intravenous delivery of Ad-MSCs (5 × 10^6^) in the rat model is beneficial for acute RILF treatment, as evidenced by its anti-inflammation, anti-fibrotic and anti-apoptotic effects, and good safety record^22^. Hence, in the present study, the intravenous delivery of higher cell numbers in two injections (two hours + seven days), rather than a one-time administration, was performed to maximize the therapeutic benefits with safety.

The process of fibrotic lesion formation in irradiated lungs can generally be classified into three stages: latent phase (one week post-irradiation), pneumonitic phase (2–16 weeks post-irradiation), and fibrotic phase (24 weeks post-irradiation)^18^. However, the detailed biochemical reactions involved in the pathogenesis of RILF are complicated. Apart from the ionizing irradiation that contributes to the development of pulmonary fibrosis by directly activating EMT in type II alveolar epithelial cells, fibrotic formation was largely driven by the abnormal release of fibrosis facilitators, such as TGF-β1, α-SMA, TNF-α, IL-1bβ and IL-6, after exposure to high doses of ionizing radiation^2,4,32^. TGF-β1 is a major mediator involved in pro-inflammatory responses and fibrotic tissue remodeling^12^. In fact, the present study demonstrated an acute and long-lasting increase in TGF-β1 expression in lung tissues after thoracic irradiation, and this was consistent with observations reported by Rube CE *et al*.^23^. Therefore, it is necessary to optimize the Ad-MSC transplantation time to completely prevent the appearance of both TGF-β1 peaks. In this regard, the present study demonstrated that to effectively treat RILF, the combined two doses of Ad-MSCs in the R + MSC_2h+7d_ group were effective in suppressing both TGF-β1 peaks.

It has been well-established that pro-fibrotic marker α-SMA, which directly reflect lung fibrosis, is regulated by TGF-β1 activity *via* the Smads signaling pathway^33–35^. In the present study, lower α-SMA expression levels in lung tissue sections obtained from the R + MSC_2h+7d_ group were observed, when compared to those obtained from the R + MSC_2h_ or R + MSC_7d_ groups. This was consistent with TGF-β1 levels. Previous studies have also indicated that TGF-β1 induced the differentiation of type II alveolar epithelial cells into myofibroblasts *via* the Smad pathway^9,36^. In this context, it was also observed that Ad-MSC treatment reduced the type II alveolar epithelial cell transition to the fibroblastic phenotype, and α-SMA was markedly reduced with R + MSC_2h+7d_ treatment, indicating that the double intravenous delivery of this therapy has notable inhibitory effects on fibrosis. Specifically, the delivery at two time points appeared to be optimal for the beneficial therapeutic efficacy and safety in treating RILF, which is probably through the inhibition of TGF-β1 level/activity induced by irradiation.

Inflammatory cytokines play an important role in mediating, amplifying and maintaining the RILF process^2,3^. The key regulators can be regulated by the paracrine effects and immunomodulation of MSCs^13–15^. Previous studies have shown that MSCs reduce the inflammatory response in acute lung injury models^37^. In the present study, it was observed that after irradiation, the mRNA expression levels of TNF-α, IL-1 and IL-6 were markedly reduced after R + MSC_2h+7d_ treatment. IL-2 and IL-10 are highly anti-inflammatory cytokines that inhibit macrophage production, and its treatment can attenuate acute lung injury^38^. However, gene IL-10 levels significantly increased in the R + MSC_2h+7d_ group, when compared to the other groups. Increased serum IL-10 levels after Ad-MSCs delivery may involve several mechanisms. Ad-MSCs can inhibit inflammation through autocrine IL-10 signaling^39,40^, and they can also release signals to several types of allogenic pro-inflammatory cells to alter their cytokine secretion profiles. High IL-10 levels induced by the double injection of Ad-MSCs inhibited the inflammatory process, such as neutrophil rolling, adhesion and transepithelial migration in the inflammatory host^38^.

In summary, these present results revealed that the injection of Ad-MSCs within two hours and one week of thoracic irradiation resulted in beneficial outcomes in the treatment of RILF through anti-inflammation in the pneumonitic phase and anti-fibrosis during the whole lung injury period.

Despite the potentially interesting observation and the availability of little information on the role of the double dose of Ad-MSCs in regulating alveolar epithelial cell transition into fibroblastic phenotypes in the lungs, further studies would be helpful to confirm this observation. In addition, it would also be interesting to study the direct correlation between the delivery time of Ad-MSCs and the TGF-β1 signaling pathway.

## Supplementary information

Supplementary Figure 1
