# Supplementary material for: Optimization of the adipose-derived mesenchymal stem cell delivery time for radiation-induced lung fibrosis treatment in rats
Source: Sci Rep. 2019 Apr 3;9:5589. doi: 10.1038/s41598-019-41576-5 (PMC6447528; doi:10.1038/s41598-019-41576-5)
Supplement: Supplementary file 1 — Supplementary Figure 1 [file 41598_2019_41576_MOESM1_ESM.docx]

**Supplementary Information**

**for**

**Optimization of the adipose-derived mesenchymal stem cell delivery time for radiation-induced lung fibrosis treatment in rats**

Yang Zhang,MD,^1^ Xinping Jiang,PhD,^2^ Liqun Ren,PhD^1*^

^1^Department of Experimental Pharmacology and Toxicology, Pharmaceutical Science of Jilin University, Changchun, Jilin 130021, China;

^2^Department of Oncological Radiotherapy, The First Hospital of Jilin University, Changchun 130021, China

**Corresponding author:** Liqun Ren; Department of Experimental Pharmacology and Toxicology, Pharmaceutical Science of Jilin University, 1266 Fujin Road, Changchun, Jilin, 130021, China; E-mail addresses: [renlq@ jlu.edu.cn](mailto:renlq@edu.jlu.cn)

**Supplementary Figures**

**A**


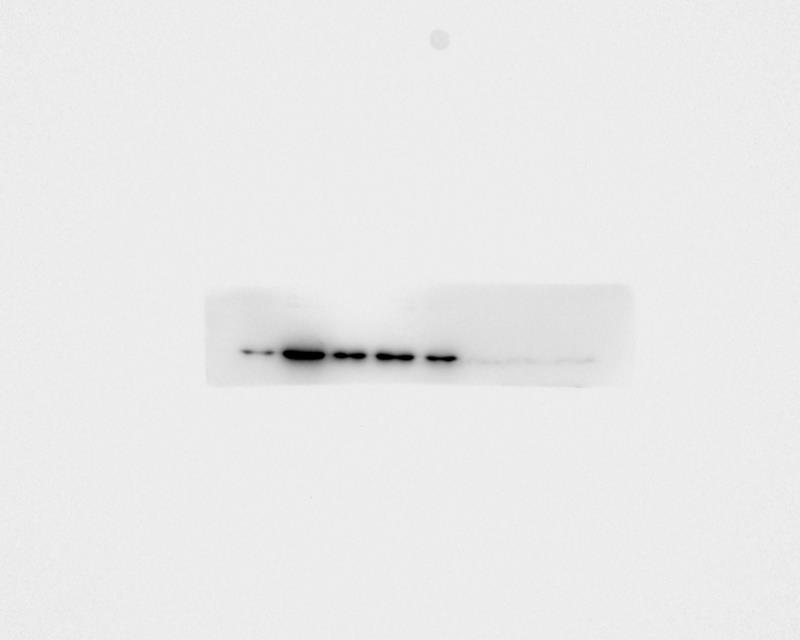


**B**


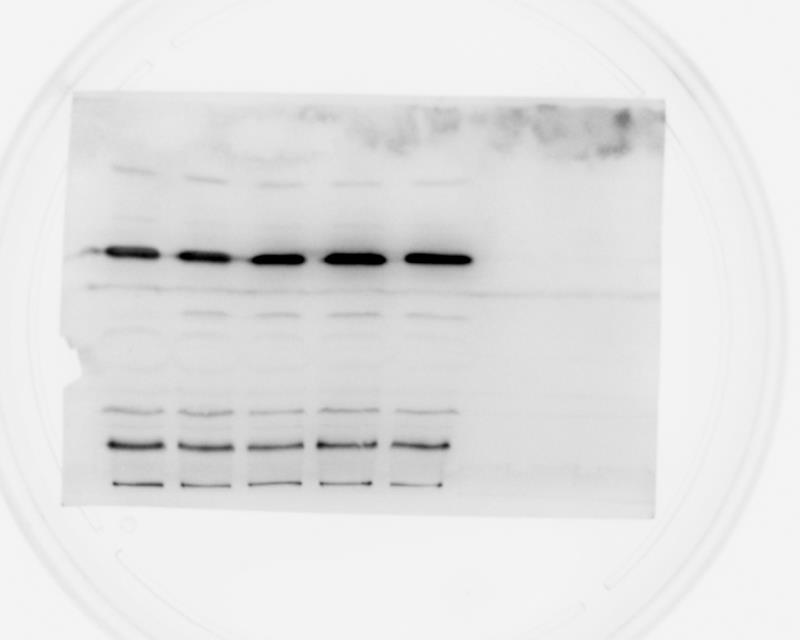


**Figure legend**

(A) The protein expression levels of α-SMA in lung tissues were analyzed by western blot after four weeks. (B) The protein expression of β-actin in lung tissues were analyzed by western blot after four weeks.
